# Watermelon (*Citrullus lanatus* (Thunb.) Matsum. and Nakai) Juice Modulates Oxidative Damage Induced by Low Dose X-Ray in Mice

**DOI:** 10.1155/2014/512834

**Published:** 2014-04-29

**Authors:** Mohd Khairul Amran Mohammad, Muhamad Idham Mohamed, Ainul Mardhiyah Zakaria, Hairil Rashmizal Abdul Razak, Wan Mazlina Md. Saad

**Affiliations:** ^1^Department of Medical Laboratory Technology, Faculty of Health Sciences, Universiti Teknologi MARA, UiTM Puncak Alam, 42300 Bandar Puncak Alam, Selangor, Malaysia; ^2^Department of Medical Imaging, Faculty of Health Sciences, Universiti Teknologi MARA, UiTM Puncak Alam, 42300 Bandar Puncak Alam, Selangor, Selangor, Malaysia

## Abstract

Watermelon is a natural product that contains high level of antioxidants and may prevent oxidative damage in tissues due to free radical generation following an exposure to ionizing radiation. The present study aimed to investigate the radioprotective effects of watermelon (*Citrullus lanatus* (Thunb.) Matsum. and Nakai) juice against oxidative damage induced by low dose X-ray exposure in mice. Twelve adult male ICR mice were randomly divided into two groups consisting of radiation (Rx) and supplementation (Tx) groups. Rx received filtered tap water, while Tx was supplemented with 50% (v/v) watermelon juice for 28 days *ad libitum* prior to total body irradiation by 100 **μ**Gy X-ray on day 29. Brain, lung, and liver tissues were assessed for the levels of malondialdehyde (MDA), apurinic/apyrimidinic (AP) sites, glutathione (GSH), and superoxide dismutase (SOD) inhibition activities. Results showed significant reduction of MDA levels and AP sites formation of Tx compared to Rx (*P* < 0.05). Mice supplemented with 50% watermelon juice restore the intracellular antioxidant activities by significantly increased SOD inhibition activities and GSH levels compared to Rx. These findings may postulate that supplementation of 50% watermelon (*Citrullus lanatus* (Thunb.) Matsum. and Nakai) juice could modulate oxidative damage induced by low dose X-ray exposure.

## 1. Introduction

A variety of highly reactive chemical entities known as reactive oxygen species (ROS) are produced by respiring cells as a small amount of the consumed oxygen is reduced [1]. ROS has dual roles, in which it can be beneficial and/or deleterious [2]. In normal biological system, the cellular functions depend on redox balance which may be defined as reduction and oxidation of prooxidants and antioxidants [2, 3]. Any distortion in the redox balance may promote oxidative stress and lead to a series of pathological condition [4].

X-ray has been clinically used as diagnostic and therapeutic tools [5]. Despite its usefulness, X-ray may also induce direct or indirect harmful effects on cellular constituents and deoxyribonucleic acid (DNA) [6, 7]. X-ray has a high penetrating power due to its low linear energy transfer (LET) and exposure to X-ray could result in generation of free radicals through radiolysis process [8]. When these free radicals interact with biological molecules, it may cause cellular lipid peroxidation and DNA damage [9].

Lipid peroxidation can be defined as the oxidative deterioration of lipids containing carbon-carbon double bonds that yield a large number of toxic byproducts [10]. Membrane lipids are highly susceptible to free radical damage [11]. The highly damaging chain reaction occurs as the lipids react with free radicals and this can lead to a production of various end products including malondialdehyde (MDA), the main carbonyl compound [11, 12]. Free radicals especially hydroxyl radicals react with DNA molecules through several mechanisms producing a broad spectrum of structural damage [13, 14]. These structural DNA damages include oxidative base modification, single strand break (SSB), double strand break (DSB), cross-links, clustered base damage, and mismatch repair (MMR) that may affect the cell's ability to transcribe the genes which are encoded by affected DNA [13].

An antioxidant is known as a molecule that acts as free radical scavenger and protects the body from oxidative damage [15]. A study by Srinivasan et al. [16] reveals that an antioxidant defense mechanism is applied to maintain redox balance, and appropriate antioxidants may reduce the free radical toxicity and protect from radiation damage [17]. Defense mechanism such as superoxide dismutase (SOD) is responsible for catalyzing the dismutation of the superoxide anion (O_2_^−^) into oxygen and hydrogen peroxide (H_2_O_2_) [18], while glutathione (GSH) provides protection against oxidative damage by participating in the cellular defense system and its intracellular level may be assessed as an indicator of oxidative stress [19].

The dietary guidelines recommended by A. V. Rao and L. G. Rao [20] suggest to increase the consumption of plant-based food that are rich in carotenoids, a bright coloured microcomponent, which is present in fruits and vegetables. Watermelon (*Citrullus lanatus*) contains a high level of carotenoids such as lycopene, beta-cryptoxanthin, beta-carotene, and vitamin E and it is proven to scavenge free radicals [21].* Citrullus lanatus* (Thunb.) Matsum. and Nakai is the most polymorphic among all* Citrullus* species which has wild, cultivated, and feral forms [22]. Altaş et al. [23] demonstrate that the nature of chemicals present in watermelon is responsible for the reduction of lipid peroxidation.

Thus, the aim of this study was to evaluate the antioxidant capacity of watermelon juice and its protective effect on low dose X-ray-induced oxidative damage in mice model.

## 2. Materials and Methods

### 2.1. Chemicals

Oxiselect Total Glutathione Assay Kit, Oxiselect TBARS Assay Kit (MDA Quantification), Oxiselect Superoxide Dismutase Activity Assay, and Oxiselect Oxidative DNA Damage (AP Sites) were purchased from Cell Biolab, Inc. (San Diego, CA), while Invisorb Spin Tissue Mini Kit was purchased from Stratec Molecular (Berlin, Germany).

### 2.2. A 50% (v/v) Watermelon Juice Preparation

A locally harvested, red seedless, watermelon juice was freshly prepared on a daily basis. The watermelon was cleaned with filtered tap water and peeled to obtain the red flesh. The flesh was then processed with a commercial juice maker which automatically separated the pulp and the juice. A 50% concentration was prepared by diluting a pure watermelon juice with filtered tap water in the ratio of 1 : 1 (v/v).

### 2.3. Animal Handling and Study Design

All animal studies were conducted in accordance with the criteria of the investigations and Universiti Teknologi MARA Committee of Animal Research and Ethics (UiTM CARE) guidelines concerning the use of experimental animals.

Twelve, healthy, four-week-old male ICR mice, each weighing about 30 grams, were obtained from Laboratory Animal Facility and Management (LAFAM), Faculty of Pharmacy, UiTM Puncak Alam Campus. The animals underwent acclimatization period for 14 days and normal mouse diet along with filtered tap water was given* ad libitum*.

The study involved two groups of seven-week-old male ICR mice and each weighting 31.3 grams which consisted of radiation group (Rx) and watermelon juice supplementation group (Tx) with six animals in each group. Mice from Tx were supplemented with 50% watermelon juice as the sole liquid source* ad libitum *for 28 days, while the Rx were only given filtered tap water. All the mice were fed with normal mouse diet. Watermelon juices were changed twice/day and the volume of watermelon juice consumed by each mouse was recorded. On day 29, both groups were exposed to a total body irradiation of a single dose X-ray.

### 2.4. Irradiation and Tissues Collection

Both groups were placed in cages under Philips Bucky DIAGNOST X-ray machine and treated with single fractionated of 100 *μ*Gy X-ray for total body irradiation. This low dose irradiation was performed by a qualified radiographer at Medical Imaging Laboratory, Faculty of Health Sciences, UiTM Puncak Alam Campus. All the mice were sacrificed by cervical dislocation within 12 hours following total body irradiation. The brain, lung, and liver tissues were excised immediately and stored at −80°C prior to analysis.

### 2.5. Lipid Peroxidation Product, MDA Assay

Tissue samples were resuspended at 100 mg/mL in PBS containing 1X butylated hydroxytoluene (BHT). Five grams of the tissue samples was homogenized on ice, spun at 10,000 g for five min. The supernatant was collected and assayed directly for its TBARS level. MDA in samples and standards was interacted with thiobarbituric acid (TBA) at 95°C and incubated and then read spectrophotometrically at 532 nm with POLARstar Omega Reader. MDA levels were determined by comparison with predetermined MDA standard curve.

### 2.6. Oxidative DNA Damage (AP Sites)

Genomic DNAs of brain, lung, and liver were isolated with Invisorb Spin Tissue Mini Kit (Stratec Molecular, Berlin) following the manufacturer's protocol. DNA Damage Quantification Kit (AP Sites) was used to quantitate apurinic/apyrimidinic (AP) sites in tissue of interest. The aldehyde reactive probe (ARP) that reacts specifically with an aldehyde group on the open ring form of AP sites (ARP-derived DNA) was detected with Streptavidin-Enzyme Conjugate. The quantity of AP sites in unknown DNA sample of brain, lung, and liver was determined using POLARstar Omega Reader at 450 nm by comparing standard curve of predetermined AP sites. All unknown DNA samples and standard were assayed in duplicate.

### 2.7. SOD Activity Assay

The activity of SOD was determined by using Oxiselect Superoxide Dismutase Activity Assay. Tissues were homogenized on ice using mortar and pestle in 7 mL of cold 1X Lysis Buffer per gram tissue followed by centrifugation at 12000 ×g for 10 minutes. The supernatant of tissue lysate was then collected and kept at −80°C until further analysis. Superoxide anions generated by Xanthine/Xanthine Oxidase system were detected with a Chromagen Solution by measuring the absorbance reading at 490 nm using POLARstar Omega Reader. The activity of SOD was determined as the inhibition percentage of chromagen reduction.

### 2.8. GSH Antioxidant Assay

Tissues were blot-dried and weighed. Ice-cold 5% metaphosphoric acid (MPA) was added and homogenized using mortar and pestle and then centrifuged at 12,000 rpm for 15 min at 4°C. The supernatant was collected. The levels of GSH were measured kinetically with a spectrophotometric kit (Oxiselect Total Glutathione Assay) according to the manufacturer's protocol. The chromogen that reacted with the thiol group of GSH produced colored compound which was then detected with POLARstar Omega Reader at 405 nm. The total GSH content in the samples was determined by comparison with GSH standard curve.

### 2.9. Statistical Analysis

All mean ± SEM (standard error of mean) values were calculated and statistical analysis was done using SPSS version 18.0 (SPSS Inc., Chicago, IL, USA) for Windows. Data were analyzed by one-way analysis of variance (ANOVA), followed by* post hoc* Tukey test for multiple comparison of mean. The difference was considered significant when *P* value was less than 0.05 (*P* < 0.05).

## 3. Results

### 3.1. Dietary Supplementation of 50% Watermelon (*Citrullus lanatus* (Thunb.) Matsum. and Nakai) Juice Conferred Remarkable Radioprotection against Lipid Peroxidation

**Figure 1:**
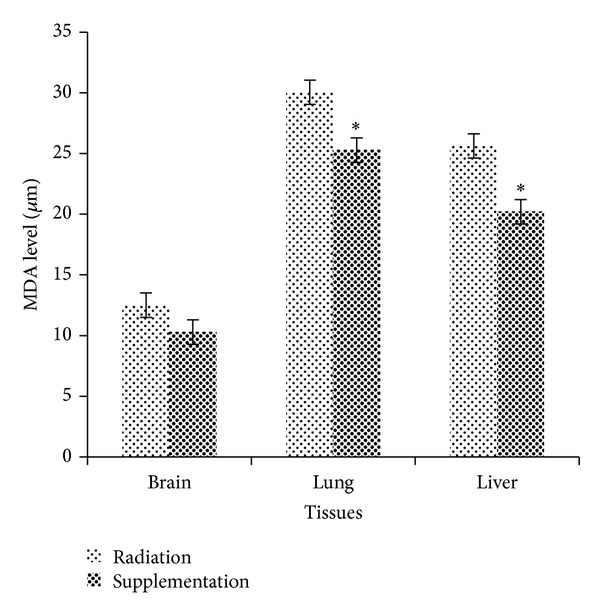
Radioprotective effects of 50% watermelon (*Citrullus lanatus *(Thunb.) Matsum. and Nakai) juice against lipid peroxidation in mice tissues. The bar chart shows the levels of MDA in brain, lung, and liver tissues of Rx and Tx. Values were expressed as mean ± SEM (*n* = 6). *Significant difference between Rx and Tx (*P* < 0.05).

The results obtained from the experimental analysis of MDA levels in mice brain, lung, and liver tissues are presented in Figure 1. There was no significant reduction of MDA levels in brain tissues of Tx compared to Rx. However, MDA levels in lung and liver tissues of Tx were significantly reduced compared to Rx with *P* = 0.004 and *P* = 0.01, respectively. The average MDA levels in lung tissues of Tx and Rx were 25.28 ± 0.45 *μ*M and 30.05 ± 0.94 *μ*M, respectively, while the average MDA level in liver tissues of Tx was 20.20 ± 0.73 *μ*M and Rx was 25.63 ± 1.43 *μ*M.

### 3.2. Dietary Supplementation of 50% Watermelon (*Citrullus lanatus* (Thunb.) Matsum. and Nakai) Juice Conferred Remarkable Radioprotection against Oxidative DNA Damage by Mitigating Number of AP Sites

**Figure 2:**
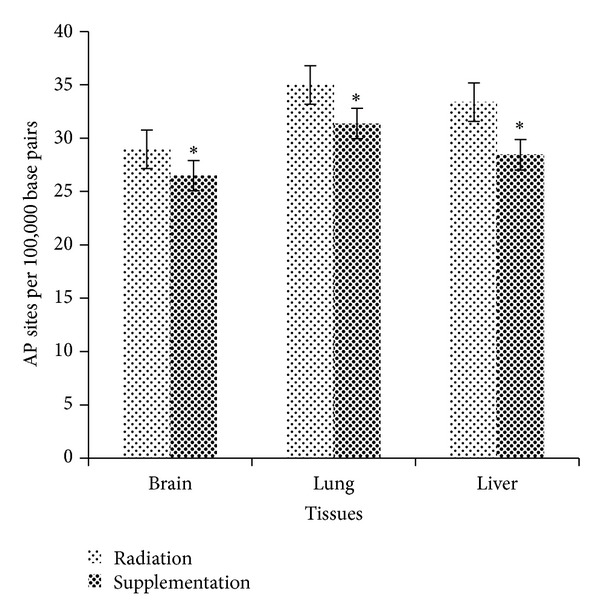
Radioprotective effects of 50% watermelon (*Citrullus lanatus *(Thunb.) Matsum. and Nakai) juice against oxidative DNA damage (AP sites). The bar chart shows the number of AP sites per 10^5^ base pairs in brain, lung, and liver tissues of Rx and Tx. Values were expressed as mean ± SEM (*n* = 6). *Significant difference between Rx and Tx (*P* < 0.05).

The radioprotective effects of 50% watermelon [*Citrullus lanatus* (Thunb.) Matsum. and Nakai] juice against oxidative DNA damage (AP sites) in mice tissues are shown in Figure 2. The generation of noncoding AP sites in brain showed significant differences between Tx and Rx with *P* = 0.029. The average numbers of AP sites per 10^5^ base pairs in Tx and Rx were 26.48 ± 0.81 and 28.96 ± 0.43, respectively. As shown in Figure 2, lung DNA revealed significantly reduced number of abasic sites per 10^5^ base pairs in Tx (31.38 ± 0.58) compared to Rx (34.98 ± 0.73) with *P* = 0.018. Meanwhile, the number of AP sites generated per 10^5^ base pairs in liver of Tx showed significantly reduced number compared to Rx (*P* = 0.05). The average AP sites generated in Tx and Rx were 28.44 ± 1.17 and 33.37 ± 0.94, respectively.

### 3.3. Dietary Supplementation of 50% Watermelon (*Citrullus lanatus* (Thunb.) Matsum. and Nakai) Juice Conferred Remarkable Radioprotection against Oxidative Stress by Mitigating SOD Activities

**Figure 3:**
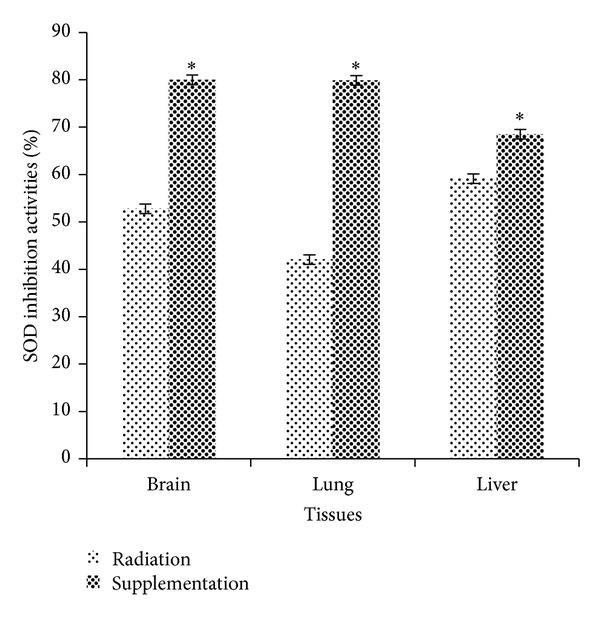
Radioprotective effects of 50% watermelon (*Citrullus lanatus *(Thunb.) Matsum. and Nakai) juice on SOD activities. The bar chart shows the percentage SOD inhibition activity in mice brain, lung, and liver tissues of Rx and Tx. Values were expressed as mean ± SEM (*n* = 6). *Significant difference between Rx and Tx (*P* < 0.05).

Figure 3 referred to the mean value of SOD inhibition activities in Tx and Rx for mice brain, lung, and liver tissues. There were significant differences between brain SOD inhibition activities in Tx (80.02 ± 1.69%) and Rx (52.79 ± 2.03%) with *P* = 0.001. Meanwhile, SOD activities increased significantly in lung tissues of Tx compared to Rx (*P* = 0.001). The average SOD activities in lung tissues of Tx and Rx were 79.90 ± 1.91% and 42.06 ± 1.24%, respectively. In liver tissue, there was a significant difference between Tx (68.50 ± 1.82%) and Rx (59.13 ± 2.0%) with *P* = 0.04.

### 3.4. Dietary Supplementation of 50% Watermelon (*Citrullus lanatus* (Thunb.) Matsum. and Nakai) Juice Conferred Remarkable Radioprotection against Oxidative Stress by Mitigating GSH Levels

**Figure 4:**
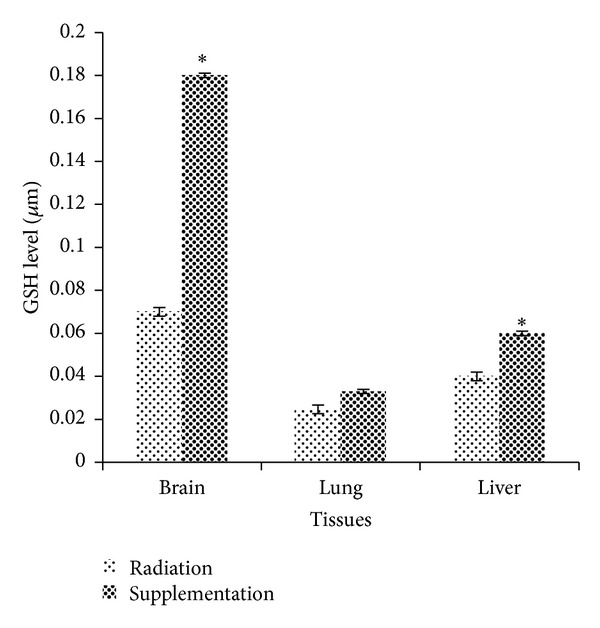
Radioprotective effects of 50% watermelon (*Citrullus lanatus *(Thunb.) Matsum. and Nakai) juice on GSH levels. The bar chart shows the GSH levels in brain, lung, and liver tissues in Rx and Tx. Values were expressed as mean ± SEM (*n* = 6). *Significant difference between Rx and Tx (*P* < 0.05).

Figure 4 shows the levels of GSH content in mice brain, lung, and liver tissues. In the present study, GSH levels in brain tissues of Tx (0.18 ± 0.0085 *μ*M) showed significant increment compared to Rx (0.07 ± 0.006 *μ*M) with *P* = 0.001. GSH levels in lung tissues of mice supplemented with watermelon juice (Tx) increased compared to Rx but no significant differences (*P* > 0.05) were observed. However, GSH levels in liver tissues of Tx were significantly increased compared to Rx (*P* = 0.003). The average GSH levels in Tx and Rx were 0.06 ± 0.001 *μ*M and 0.04 ± 0.002 *μ*M, respectively.

## 4. Discussion

Oxygen radicals react with PUFA residues in phospholipids resulting in end products that are mostly reactive towards protein and DNA. One of the most abundant carbonyl products of lipid peroxidation is MDA [24]. Low dose X-ray might cause lipid peroxidation and the finding of this present study has shown that mice supplemented with 50% watermelon juice (Tx) resulted in a marked reduction in MDA levels in lung and liver tissues compared to Rx (Figure 1). Supplementation with 50% watermelon juice restored the activities of intracellular antioxidant enzymes in mice lung and liver tissues following exposure to low dose X-ray. Thus, phytochemical antioxidants contents in 50% watermelon juice may possibly contribute to the efficacy of intracellular antioxidant defense system by providing a puissant consumer of free radicals which induced oxidative damage. This was in agreement with the study conducted by Asita and Molise [1] which reveals that watermelon contains higher content of carotenoids such as lycopene and has proven to scavenge free radicals thus inhibit lipid peroxidation.

DNA continuously generates sites of missing bases termed as abasic or apurinic/apyrimidinic (AP) sites through exposure to endogenous and exogenous sources which are capable to induce oxidative DNA damage [25]. This study demonstrated that low dose X-ray exposure induced oxidative DNA damage was indeed positively correlated with AP sites formation. Here, these results show that mice supplemented with 50% watermelon juice in the presence of low dose X-ray exposure significantly prevented progressive increase of AP sites formation in brain, lung, and liver tissues compared to mice irradiated with low dose X-ray alone (Figure 2). It is seen possible to suggest that these results are mainly due to synergistic interaction between micronutrients content in watermelon juice and intracellular antioxidant enzymes could modulate oxidative DNA damage induced by low dose X-ray exposure. The present finding seems to be consistent with a previous study by Shokrzadeh et al. [26] which showed that mice preadministered with* Citrullus colocynthis* (L.) extract or locally known as watermelon for seven consecutive days via intraperitoneal injection followed by injection with 70 mg/kg body weight of cyclophosphamide- (CP-) induced DNA damage significantly reduced the number of micronucleated polychromatic erythrocytes (MnPCEs), an index of oxidative DNA damage.

SOD plays an important role in reducing the effect of free radicals attack, and SOD is the only enzymatic system quenching O_2_^−^ to oxygen and H_2_O_2_ and plays a significant role against oxidant stress [18]. Referring to Figure 3, the percentage of SOD inhibition activities in brain, lung, and liver tissues of Tx showed significant increment compared to Rx. It seems possible to suggest that these results are mainly due to watermelon containing high level of phytonutrients including lycopene [27]. Perkins-Veazie et al. [28] point out that lycopene is a highly effective antioxidant because it acts as a strong free radical scavenger compared to carotenoids including beta-carotene, alpha carotene, lutein, beta-cryptoxanthin and astaxanthin in biological systems. In this context, the micronutrient antioxidant contents, especially lycopene, in 50% watermelon (*Citrullus lanatus* (Thunb.) Matsum. and Nakai) juice, accumulate in the tissues and counteract the deleterious effects of free radicals generated by low dose X-ray through activation of oxygen molecules.

GSH has been reported to have protective roles against oxidative stress through scavenging hydroxyl radical and singlet oxygen directly detoxifying H_2_O_2_ and lipid peroxides and also regenerate important antioxidants, Vitamins C and E, back to their active forms [2]. In the present study, the GSH levels in brain and liver tissues of Tx showed significant increment compared to Rx but no significant increment in lung. This phenomenon may suggest that the supplementation of antioxidant in 50% watermelon juice has successfully elevated the levels of GSH in both brain and liver tissues. Present results were in line with a study by Saada et al. [29] which emphasized that pretreatment with lycopene, which is rich in watermelon, significantly improved the oxidant/antioxidant status and helped in reducing oxidative damage due to radiation.

## 5. Conclusion

This study clarifies that the supplementation of 50% watermelon juice possesses benefits in modulating the oxidative damage induced by low dose X-ray exposure in terms of suppressing the levels of MDA and noncoding AP sites formation while enhancing the levels of SOD and GSH activities.
